# Adaptive Crack Modeling with Interface Solid Elements for Plain and Fiber Reinforced Concrete Structures

**DOI:** 10.3390/ma10070771

**Published:** 2017-07-08

**Authors:** Yijian Zhan, Günther Meschke

**Affiliations:** 1 Institute for Structural Mechanics, Ruhr University Bochum, Universitätsstraße 150, 44801 Bochum, Germany; yijian.zhan@rub.de; 2 Shanghai Construction Group Co., Ltd., Shanghai 200080, China

**Keywords:** fiber-reinforced concrete, crack model, interface solid element, finite element method, mesh adaptation, computational efficiency

## Abstract

The effective analysis of the nonlinear behavior of cement-based engineering structures not only demands physically-reliable models, but also computationally-efficient algorithms. Based on a continuum interface element formulation that is suitable to capture complex cracking phenomena in concrete materials and structures, an adaptive mesh processing technique is proposed for computational simulations of plain and fiber-reinforced concrete structures to progressively disintegrate the initial finite element mesh and to add degenerated solid elements into the interfacial gaps. In comparison with the implementation where the entire mesh is processed prior to the computation, the proposed adaptive cracking model allows simulating the failure behavior of plain and fiber-reinforced concrete structures with remarkably reduced computational expense.

## 1. Introduction

Concrete is one of the most important construction materials due to its well-recognized advantages, such as low cost, easy moldability and high strength under compression. However, unreinforced concrete exhibits a quasi-brittle behavior and, therefore, needs to be strengthened under tension-dominant loading conditions. As compared to conventional steel-reinforced concrete, fiber-reinforced concrete (FRC), characterized by high post-cracking ductility or even strain-hardening behavior accompanied by distributed cracks at a small crack width, can be employed to control the development of localized cracks particularly in local areas such as concrete cover and corner regions. Modern FRC has experienced a fast development since the 1960s, leading to a variety of FRC material designs with different fiber materials, geometries and performance (see, e.g., [1] for an overview). FRC is playing an increasingly important role in structural engineering as it partially or fully replaces traditional reinforced concrete. An example for a complete replacement of reinforcing steel is the design of segmented tunnel linings made of FRC [2].

In the last few decades, the material properties, as well as the structural performance of plain and fiber-reinforced concrete have been extensively investigated in laboratory environments. However, experiments are in general expensive and are limited to specific test configurations. Therefore, a variety of numerical models for concrete cracking, aiming at reliable prognoses of the fracture processes of concrete structures with or without reinforcement, have been proposed (see, e.g., [3,4,5,6,7,8] for an overview). The majority of models for structural analyses of FRC are conceptually based on cracking models for plain concrete, modifying the post-peak regime of the constitutive law in terms of an increase of the residual stress and the fracture energy, so as to represent the enhanced ductility of FRC at a phenomenological level [9,10,11,12]. To enable the analysis of the influence of specific fiber cocktails on the macroscopic material behavior of FRC, computational meso-scale models for FRC have been proposed, which include the explicit description of individual fibers within representative elementary volumes of FRC samples [13,14,15]. However, for computational analyses on a structural level, a multiscale-oriented approach allowing one to formulate the behavior of fiber and matrix and their mutual interactions at different length scales as proposed in [16] is required. Recently, the authors proposed a multilevel modeling framework, in which, at the lowest scale, the pullout behavior of different fiber types interacting with the concrete matrix at different inclination angles is considered by appropriate sub-models with the model information being appropriately transmitted across the scales depending on the specific fiber cocktail [17].

As an essential component of the modeling framework presented in [17], interface solid elements (ISE), i.e., degenerated solid finite elements with almost zero thickness proposed by Manzoli et al. [18,19,20,21], have been adopted and successfully applied to the failure analysis of plain and fiber-reinforced concrete structures. As compared with classical zero-thickness interface elements, ISE can be easily implemented based on standard finite element codes by using solid finite elements for the bulk material and for the interfaces. Employing a continuum damage model to approximate the interface degradation, it allows one to describe the interface behavior completely in the continuum framework. Consequently, those specific variational formulations, discrete constitutive relations and integration rules to obtain the internal forces associated with classical interface elements are not required. The artificial initial stiffness that is normally required in zero-thickness interface elements is automatically included in the elastic stiffness of ISE [18,20,21]. It is recognized that the interface solid elements share similar features with zero-thickness interface elements. The most notable advantage of this class of models is the fact that no special procedure for the tracking of evolving cracks is necessary. This contributes to its robustness and allows for 3D fracture simulations characterized by complex fracture patterns (see, e.g., [22]). The crack pattern obtained via discrete representations along prescribed element edges evidently suffers from a certain dependence on the mesh topology. However, the influence on the overall macroscopic material response is tolerable if unstructured meshes with reasonable resolution are used [20,23]. Furthermore, for analyses of heterogeneous materials on the mesoscale level, it was shown that the mesh-dependence of interface elements becomes less of a concern once the mesoscale heterogeneity is modeled [24,25,26]. This drawback can be alleviated, e.g., by continuously modifying the local finite element topology at the crack tip to enforce the alignment between the element edges and the crack propagation direction [27,28,29]. Alternatively, mesh refinement, at the cost of increased computational expense, can be applied to resolve the large elements along the crack path [30,31,32]. The increased computational demand resulting from the duplication of finite element nodes can be controlled by pre-defining the interface elements only in vulnerable regions or applying an adaptive algorithm for the mesh processing during computation [33,34,35,36,37].

As unstructured, high density finite element discretizations are required in general to reduce the influence of the mesh on the computed crack topology, we propose an adaptive crack model for plain and fiber-reinforced concrete structures, in which the mesh is progressively modified during the analysis as fracture proceeds. It is based on a previous implementation of the ISE technology, where the complete finite element mesh was preprocessed for the insertion of interface solid elements [17]. Solid elements are generated within interfacial gaps only in the vicinity of the crack path to allow for structural analysis with significantly reduced computational costs. The proposed numerical methods are designed to provide a platform for physically-sound and computationally-efficient and robust numerical analyses of concrete and (fiber or steel) reinforced concrete structures. However, the focus of this paper is specifically on the numerical modeling and analysis of fiber-reinforced materials and structures using adaptive strategies for inserting interface solid elements.

## 2. Multilevel Model for Fiber-Reinforced Concrete

A multilevel model for steel fiber-reinforced concrete materials and structures, which allows one to follow the influence of various design parameters through the different length scales [17], is briefly summarized in this section. The model consists of a series of model components associated with three different scales involved in the numerical analysis of FRC:

- Level 1: Modeling of the pullout behavior of single fibers;
- Level 2: Modeling of the crack bridging effect of fiber cocktails;
- Level 3: Structural simulation including the opening and propagation of cracks considering the fiber crack bridging effect.

### 2.1. Level 1: Single Fiber Pullout Model

**Figure 1:**
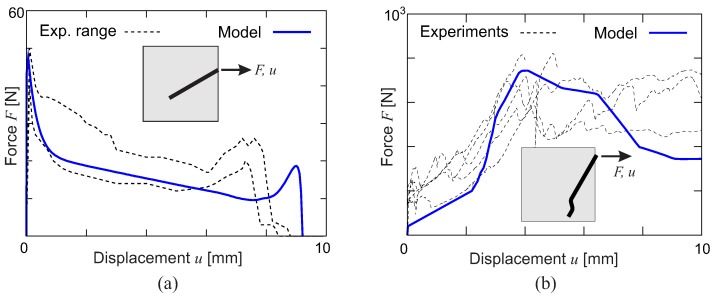
Selected results of the single fiber pullout model [38]: (**a**) straight steel fiber with a 30${}^{\circ }$ inclination angle with respect to the loading direction; (**b**) hooked-end steel fiber with a 60${}^{\circ }$ inclination angle.

At the level of the interaction between individual fibers and the matrix (Level 1), the pullout behavior of a single fiber is controlled by interface conditions, the fiber shape and the fiber inclination with respect to a crack. An analytical model for single fiber pullout has been proposed [38]. This model is able to capture the major mechanisms involved during the pullout of a single steel fiber embedded in a concrete matrix, accounting for different configurations of fiber type and strength, concrete strength and fiber inclination, as demonstrated in Figure 1.

### 2.2. Level 2: Crack Bridging Model

At the level of an opening crack within the fiber-concrete composite (Level 2), using the analytical model for single fiber pullout, the bridging effect, i.e., the traction (*t*) vs. the separation (*w*) law, is obtained via the integration of the pullout response of all fibers intercepting the crack.

**Figure 2:**
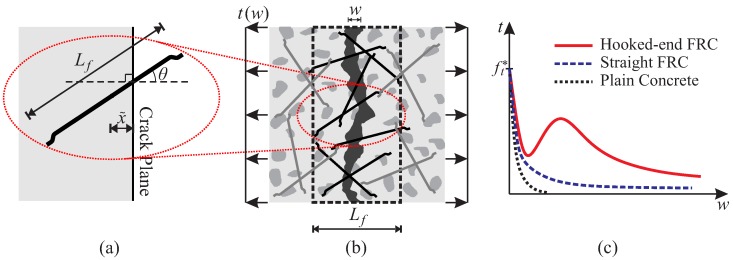
Crack bridging model: (**a**) position and inclination of a fiber with respect to the crack; (**b**) unit area of an opening crack in FRC intercepted by fibers with length $L_{f}$; (**c**) sketch of the obtained traction-separation relations for different FRC composites.

A unit area of an opening crack and the corresponding volume element of the composite material, containing a number of distributed fibers crossing the crack, is considered (Figure 2b). The pullout response of each fiber is dependent on the position $\tilde{x}$ and the inclination *θ* of the fiber with respect to the crack plane (Figure 2a). As the crack opens (*w* increases), the integration of the individual fiber pullout forces with respect to the position and inclination results in the fiber bridging stress [39]:

(1) $$t^{\mathrm{fib}}\left(w\right)=\frac{c_{f}}{A_{f}}\int_{\tilde{x}=0}^{L_{f}/2}\left[\int_{\theta =0}^{arccos(2\tilde{x}/L_{f})}F\left(\tilde{x},\theta ,w\right)\;p\left(\theta \right)\;d\theta \right]p\left(\tilde{x}\right)\;d\tilde{x}.$$

In Equation (1), $c_{f}$ is the volume fraction of the fibers, and $A_{f}$ is the cross-section area of one fiber. The single fiber pullout force $F(\tilde{x},\;\theta ,\;w)$ is dependent on $\tilde{x}$ and *θ*. The spatial dispersion characteristics $p(\theta ,\;\tilde{x})$ of the fiber cocktail in the composite are represented by the probability density *p* as a function of the inclination angle *θ* and the position $\tilde{x}$ of the fiber. While performing the integration in the present model, the following parameters influencing the evaluation of p(θ) and $p(\tilde{x})$ and, consequently, the bridging effect are particularly taken into account [17]:

- Fiber orientation: Unlike the usual assumption of the isotropic spatial orientation of fibers (see, e.g., [39]), an anisotropic fiber orientation as a general result of the casting process, graphically represented as an ellipsoid, is assumed to represent the spatial preference of the fiber cocktail in the global coordinate system. From this ellipsoidal fiber orientation, in association with a given (or potential) crack plane, the probability density p(θ) can be obtained by computing the differential volume of the ellipsoid corresponding to a specific value of *θ*.
- Boundary effect: In the vicinity of boundaries, the fibers tend to orient parallel to the boundary surfaces [40]. This “boundary effect”, dependent on the fiber length and the dimension of the mold, is considered by means of “scanning” the potential crack plane and excluding the impossible fiber orientations according to the distance to the boundary. As a result, an average $\bar{p}\left(\theta \right)$ relation is obtained and used in Equation (1) for the calculation of the crack bridging tractions.
- The fiber distribution $p(\tilde{x})$ with respect to the position is assumed to be homogeneous [39].
- Group effect: An additional aspect to be considered is the experimental observations that only 50–90% of hooked-end steel fibers are active due to the “group effect” of fibers in the composite material. In the present model, the activity factor is generally assumed to be 70%.

**Figure 3:**
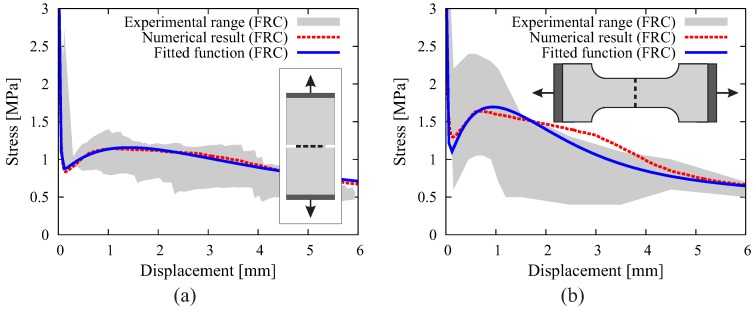
Results of crack-bridging effect in FRC obtained from the investigation of uniaxial tension tests on (**a**) a notched prism cast in a “standing” mold and (**b**) a “dog bone” specimen cast in a “lying” mold.

The bridging effect for an opening crack is obtained as the total contribution of the cohesive traction of concrete and the contribution of the fiber cocktail. As the determination of $t^{fib}\left(w\right)$ according to Equation (1) requires a separate computational evaluation, which would slow down the finite element analysis if directly incorporated, the numerically-obtained results for the t−w relationship are replaced by an analytical surrogate function form to be employed in the finite element structural model:

(2) $$t\left(w\right)=\left(f_{t}^{*}-t_{1}\right)\;\exp\left(-\frac{w}{w_{\mathrm{ref}}}\right)+t_{1}\frac{w_{u}-w}{w_{u}}+t_{2}w\;\exp\left(c_{1}-c_{2}w\right),$$

where $t_{1}$, $t_{2}$, $c_{1}$ and $c_{2}$ are coefficients determined from a least-square curve fitting to the original numerical evaluation of Equation (1); $w_{\mathrm{ref}}$ is a reference value of crack opening; $w_{u}=L_{f}/2$ represents the ultimate crack opening; and $f_{t}^{*}$ is the tensile strength of the FRC composite. This function form can be easily used to represent traction-separation laws for different fiber-concrete composites (Figure 2c). Figure 3 contains the validation examples demonstrating the quality of the model in describing the bridging effect of fibers across the horizontal crack in a notched FRC prism cast in a “standing” mold [41] and the vertical crack in a “dog bone” specimen cast in a “lying” mold [42].

### 2.3. Level 3: Failure Analysis of FRC Structures

**Figure 4:**
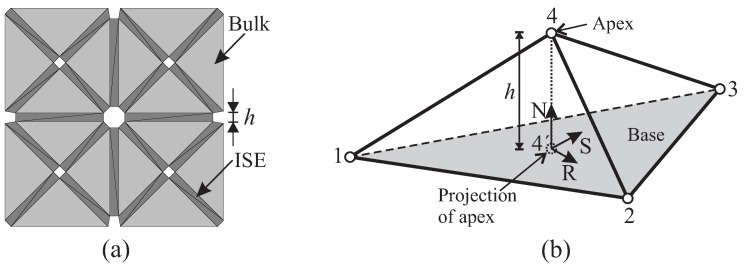
(**a**) Processed mesh for computation, obtained after the insertion of solid elements into all interfacial gaps; (**b**) degenerated 3D solid element characterized by the “base”, the “apex” Node 4 and its projection on the base (Point 4’). ISE, interface solid element.

Level 3 of the model is concerned with a finite element implementation employing “interface solid elements (ISEs)” for simulating propagating cracks. Instead of the classical zero-thickness interface elements, degenerated 2D and 3D solid elements with almost zero thickness as proposed by Manzoli et al. [18] and Sanchez et al. [20] are used. Through a pre-processing algorithm, the original mesh is entirely fragmented, and interface solid elements are generated and placed along all element edges as illustrated in Figure 4a. Using degenerated solid elements to represent cracks, the strain in these interface elements is assumed to be exclusively related to the (regularized) unbounded term $\hat{\varepsilon }$ according to the continuum strong discontinuity concept [18,20]:

(3) $$\varepsilon \approx \hat{\varepsilon }=\frac{1}{h}(〚u〛⊗n{)}^{s}.$$

In 3D, the strain tensor in an ISE can be expressed in the local coordinate system (*N*-*R*-*S*) as:

(4) $$\varepsilon \approx \frac{1}{h}\left[\begin{array}{ccc} 〚u{〛}_{n} & 〚u{〛}_{r}/2 & 〚u{〛}_{s}/2\\ 〚u{〛}_{r}/2 & 0 & 0\\ 〚u{〛}_{s}/2 & 0 & 0 \end{array}\right],$$

with $〚u{〛}_{n}$, $〚u{〛}_{r}$ and $〚u{〛}_{s}$ as the normal and tangential components of the displacement jump 〚u〛, respectively, which are determined from the relative displacement of the apex node with respect to its projection on the base (Figure 4b). Note that in the present work, only constant strain elements, i.e., three-node-triangular (in 2D analyses) and four-node-tetrahedral (in 3D analyses), are employed both for bulk and interface elements.

All bulk elements are considered to be linear elastic. The constitutive behavior of the degenerated solid elements is cast in a continuum form equipped with a damage law, which allows one to approximate the behavior of interfacial degradation mechanisms involved during the cracking in FRC materials:

(5) $$\sigma =\left(1-d\right)C_{e}:\varepsilon \approx \left(1-d\right)\frac{1}{h}C_{e}:(〚u〛⊗n{)}^{s},$$

with *d* as the scalar damage variable and $C_{e}$ denoting the elastic stiffness tensor with Poisson’s ratio ν=0. The loading criterion is defined in terms of the equivalent stress $\tilde{\sigma }$ and the displacement-like internal parameter *α*:

(6) $$f\left(\sigma ,\alpha \right)=\tilde{\sigma }-t\left(\alpha \right)\leq 0.$$

Here, $\tilde{\sigma }=\sqrt{\sigma_{n}^{2}+{\left(\sigma_{r}/\beta \right)}^{2}+{\left(\sigma_{s}/\beta \right)}^{2}}$ is considered, with the factor β representing a weighting of normal and shear contributions to account for mixed mode fracture; in the present work, β=2.0 is used [33,43,44]. The softening behavior of interface t(α) is controlled by the crack bridging law Equation (2) presented in the previous section, replacing the crack width *w* with the “internal parameter” α.

The internal parameter *α* is defined as the maximum value of equivalent separation that the interface experiences during the loading history:

(7) $$\alpha =\max\left(\tilde{u}\right)-{\tilde{u}}_{0}.$$

**Figure 5:**
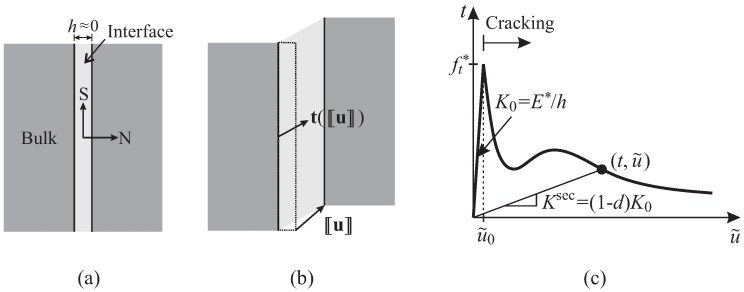
Interface in (**a**) undeformed and (**b**) deformed configuration. (**c**) Equivalent traction-separation relation for the cohesive interface model.

Here, the equivalent crack separation is defined as:

(8) $$\tilde{u}=\sqrt{〚u{〛}_{n}^{2}+{\left(\frac{〚u{〛}_{r}}{2\beta }\right)}^{2}+{\left(\frac{〚u{〛}_{s}}{2\beta }\right)}^{2}},$$

and ${\tilde{u}}_{0}$ corresponds to the elastic limit state of the interface:

(9) $${\tilde{u}}_{0}=\frac{f_{t}^{*}}{K_{0}}=\frac{hf_{t}^{*}}{E^{*}}\approx 0,$$

with $K_{0}$ representing the “rigid” elastic stiffness of the equivalent interface behavior (Figure 5).

The scalar damage variable d(α) is obtained by comparing the secant stiffness $K^{\sec}$ with the elastic stiffness of the equivalent interface behavior (Figure 5c) as:

(10) $$d\left(\alpha \right)=1-\frac{K^{\sec}}{K_{0}}=1-\frac{h\;t}{E^{*}\left(\alpha +{\tilde{u}}_{0}\right)}.$$

It is noticed that the post-cracking behavior of the FRC material is highly nonlinear; such nonlinearity frequently results in numerical difficulties while performing structural simulations. In the present work, the IMPL-EXintegration scheme [45] is implemented in the context of the interface solid element for FRC. Consequently, due to the explicit nature of damage models, the computation does not require any iteration, neither on the structural level, nor on the constitutive level. This ensures the robustness and efficiency of the computational model in failure analyses of FRC structures even in the case of complex crack configurations.

**Figure 6:**
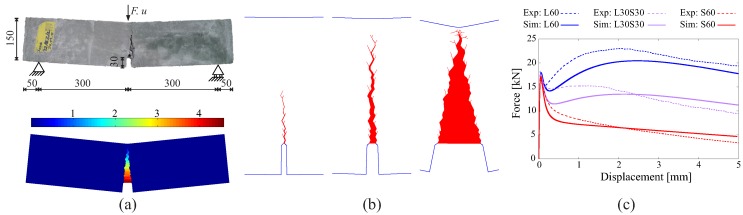
Analysis of a three-point bending test on a notched FRC beam: (**a**) photo of the failure state of the specimen and the contour plot of the crack-opening magnitude in the deformed configuration; (**b**) crack patterns represented by the activated interface solid elements at different loading states; (**c**) comparison between the force-displacement relations predicted by the proposed model and from the experiments [41] for three different fiber cocktails.

Figure 6 demonstrates the model performance while analyzing the behavior of a notched FRC beam subjected to three-point bending. It is observed that the proposed multilevel model captures the fracturing processes in FRC and the load-bearing responses of the structure both qualitatively and quantitatively very well (see Zhan and Meschke [17] for more details).

It is worth mentioning that the present work focuses on monotonic loading situations. In the case of cyclic loading, the un- and re-loading branch follow a secant in the traction-separation curve (see Figure 5c). In fact, however, residual deformations due to imperfect closing of the rough crack faces after unloading are observed already in cyclic loading of plain concrete. To appropriately represent this mechanism, it will be necessary to consider a damage-plasticity model, e.g., in a similar fashion as was proposed in [46,47], where a smooth transition from open to fully-closed cracks is considered. The irreversible deformations associated with aggregate interlocking and fiber resistance at the crack surfaces are even more pronounced in the case of FRC (e.g., [48]). However, this issue is beyond the scope of the present paper.

## 3. Mesh-Processing Techniques

### 3.1. Full Insertion of Interface Solid Elements via Preprocessing

Using interface solid elements to capture the cracking phenomena in concrete structures, it is necessary to include in the numerical implementation a code block for placing degenerated solid elements into the interfacial gaps prior to the initiation of cracks. For that purpose, a preprocessing algorithm must be executed before applying the loading conditions [18,20].

**Figure 7:**
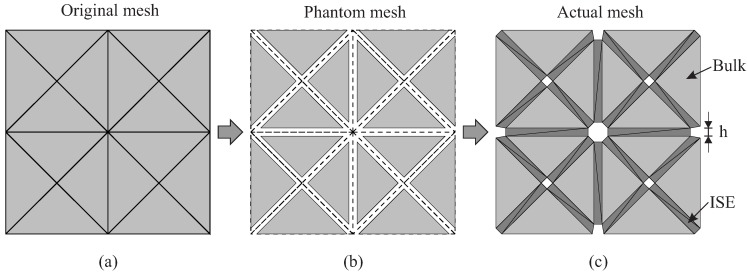
Pre-processing (insertion of interface solid elements in the complete domain): (**a**) original finite element mesh; (**b**) phantom mesh obtained by duplicating the edges and shrinking the bulk elements; (**c**) actual mesh for computation, obtained after insertion of solid elements into all interfacial gaps.

As illustrated in Figure 7, in a first step, the topology of the original finite element mesh is investigated. Based on the topological information (e.g., the neighboring bulk elements, the common edges and the node connectivity), the original mesh is fragmented. All common edges are duplicated; every bulk element shrinks via offsetting the edges by h/2; a “phantom mesh” is obtained (Figure 7b). Afterwards, every gap between the neighboring bulk elements is filled with solid elements characterized by a very high aspect ratio (Figure 7c), resulting in the actual discretization of the domain used for the computation. It is worth emphasizing that three-node-triangular (in 2D analyses) and four-node-tetrahedral (in 3D) are used both for bulk and interface elements.

In analogy to the classical zero-thickness interface elements, interface solid elements are well suited for capturing complex cracking phenomena (such as crack initiation and opening, crack propagation, kinking or even branching) in quasi-brittle materials without applying any crack path tracking technique [19]. However, it is observed from a number of structural simulations that the problem sizes (i.e., the system degrees of freedom (DOFs)) of the fully-processed mesh can reach approximately six-times (in 2D) and 20-times (in 3D analysis) the number of DOFs as compared to the original mesh. In order to limit the computational costs arising when using interface elements, different means of numerical implementation, such as performing the mesh fragmentation only in the vulnerable regions [37] and applying an adaptive algorithm for the insertion of interface elements [35], are proposed.

### 3.2. Adaptive Insertion of Interface Solid Elements

**Figure 8:**
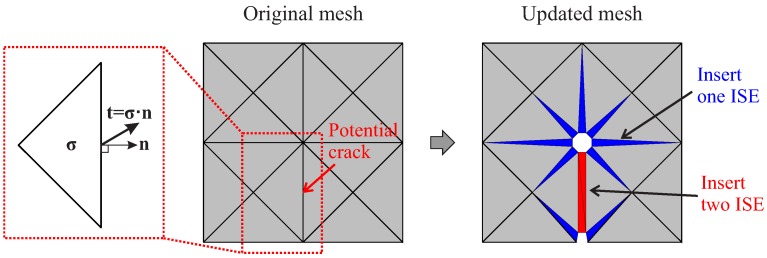
Concept of adaptive insertion of ISEs.

In the present work, a specific algorithm has been developed and implemented for the adaptive insertion of ISEs to consider potential cracks during the structural failure analysis. The general concept of the proposed algorithm is depicted in Figure 8. The adaptive insertion procedure consists of the following major steps:

1. Prior to the application of loading, use the original discretization to generate the phantom mesh.
2. Start the structural simulation with the original mesh.
3. In every load increment (except the first):
  (a) Modify the mesh by splitting the critical interfaces which were recorded at the end of the previous increment; fill the interfacial gaps with degenerated solid elements.
  (b) Solve the structural equation system based on the modified mesh.
  (c) According to the new solution, inspect the stress state in the bulk elements to identify the critical interfaces.
4. Proceed to the next increment.

It is worth pointing out that similar effort has been made for the classical zero-thickness interface elements [35]. Nevertheless, the adaptive technique has not yet been considered for the recently-proposed continuum interface elements. The details of the adaptation process are described in the following.

#### 3.2.1. Generation of the Phantom Mesh

**Figure 9:**
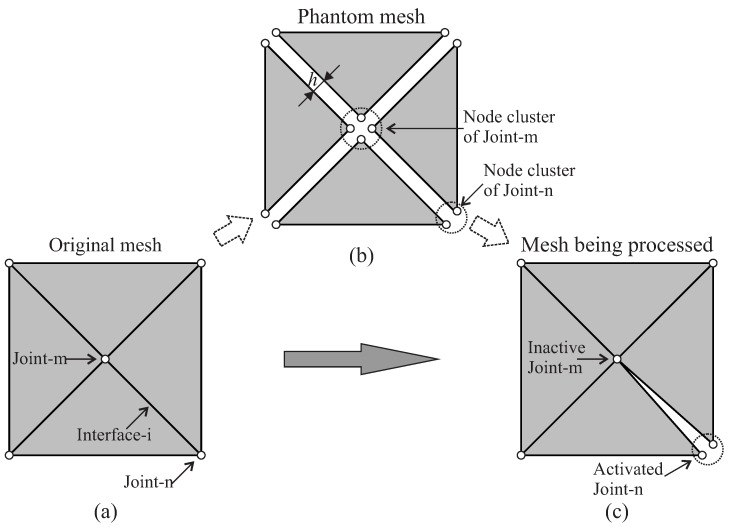
Generation and use of the phantom mesh: (**a**) original mesh; (**b**) phantom mesh; (**c**) mesh being processed (interface solid element not yet generated).

As illustrated in Figure 7, the “phantom mesh” is generated before starting the structural analysis. This process is identical for both full and adaptive insertion options. The difference lies in the subsequent steps: For the full insertion, the phantom mesh is directly used as the basis, and subsequently, solid elements are generated by correctly connecting the nodes on the two sides of the interface. However, in the adaptive algorithm, the phantom mesh provides a collection of topological information that is only partially used during simulation. As depicted in Figure 9a for a 2D configuration, every “joint” (such as joint-m or joint-n) corresponds to a node in the original mesh, and an “interface” (e.g., interface-i) connects two joints. In the phantom mesh (Figure 9b), every joint controls a cluster of phantom nodes. During the loading procedure, whenever a joint is activated, the original node (e.g., node-n) is replaced by the corresponding cluster of phantom nodes (Figure 9c).

#### 3.2.2. Determination of “Critical” Interfaces

The goal of the algorithm is to generate as few interface solid elements as possible; therefore, only the interfaces subjected to a stress level that is close to the material strength are considered as “critical”. More specifically, the traction on an interface can be calculated from the stresses (***σ***) in any of the two bulk elements:

(11) t=σ·n.

Considering an interface under tension (indicated by a positive normal component of the interface stress, i.e., $t_{n}>0$), if the magnitude of the traction vector exceeds a given threshold, this interface is denoted as critical:

(12) $$|t|>t^{\mathrm{thr}},\;\mathrm{with}\;t^{\mathrm{thr}}=\gamma \;f_{t}.$$

It should be noted that also other criteria based on, e.g., linear fracture mechanics, are alternatively used in the literature [33]. The factor 0≤γ≤1 controls the level of threshold and is generally set as 0.8, according to the authors’ experience. Setting a small value of γ, too many unnecessary interfaces may be identified as critical and too many ISEs may be generated. On the other hand, a large value (γ→1.0) may lead to the over-stressing of interfaces, since the ISE should be prepared prior to the crack initiation. With the tolerance, the value of 0.8 includes a banded region along the crack path. Consequently, the influence of the insufficient accuracy of the resolution of the stress field at the crack tip inherently obtained from using linear finite elements is eliminated.

#### 3.2.3. Mesh Adaptation: Splitting of Interfaces and the Generation of IS Elements

In order to prepare the discretized model to consider potential cracks during the analysis, the critical interfaces identified according to the criterion Equation (12) are fully separated. Since (in 2D) an interface controls two joints, the clusters of phantom nodes corresponding to both joints are activated and used. For the adjacent bulk elements, the node connectivity is updated by using the activated phantom nodes. Two ISEs filling the interfacial gap are generated based on the phantom nodes. In the meantime, the affected interfaces adjacent to this critical interface are partially split and filled with one ISE (Figure 8).

**Figure 10:**
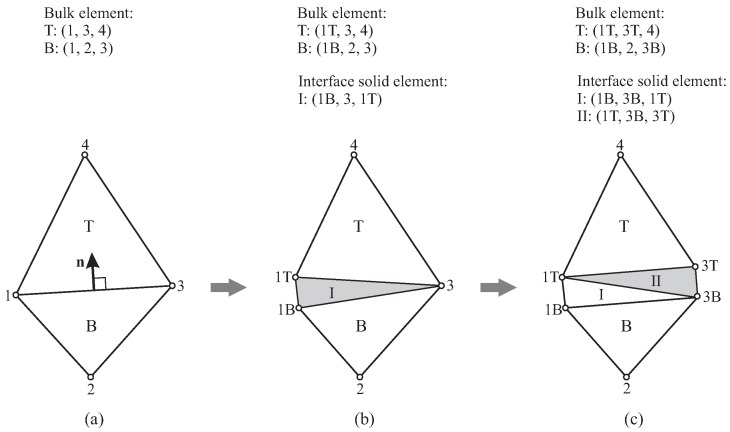
Insertion of solid elements at a specific interface in 2D: (**a**) original mesh with two bulk elements; (**b**) Node-1 split, the first interface solid element inserted (ISE-I, in gray); (**c**) Node-3 split, the second interface solid element inserted (ISE-II, in gray).

The algorithm for the insertion of ISE in a specific interface in a 2D configuration is depicted in Figure 10:

1. Initially, the interface connecting Node-1 and Node-3 is shared by two bulk elements, i.e., Element-T and Element-B. (For convenience, Element-T refers to the bulk element located on the positive side of the interface, and Element-B is on the other side; see Figure 10a).
2. As shown in Figure 10b, when Joint-1 is split, the corresponding cluster of phantom nodes (including Node-1T and Node-1B) is activated. The connectivity of bulk Element-T and Element-B is updated by replacing the current Node-1 with Node-1T and Node-1B, respectively. Meanwhile, the first interface solid element (ISE-I) is created by using Node-1T and Node-1B.
3. When Node-3 is split, activate the phantom nodes (including Node-3T and Node-3B). The nodes of bulk elements are renewed in the same way; one of the nodes of the existing ISE-I is updated, as well (by using the new Node-3B instead of the current Node-3). In addition, the second ISE (ISE-II) is generated.

Note that the procedure described above is not limited to the processing of a critical interface within one load increment. In general, there are three possible situations for the opening of a 2D interface; the other two cases are as follows: (1) An interface is partially open due to the activation of an adjacent interface; this process can be graphically represented by Figure 10a,b. (2) After a certain number of load increments, a partially split interface (Figure 10b) becomes fully separated (Figure 10c), because the interface itself or a neighboring interface reaches the critical state (as will be illustrated in the numerical examples contained in Section 4).

**Figure 11:**
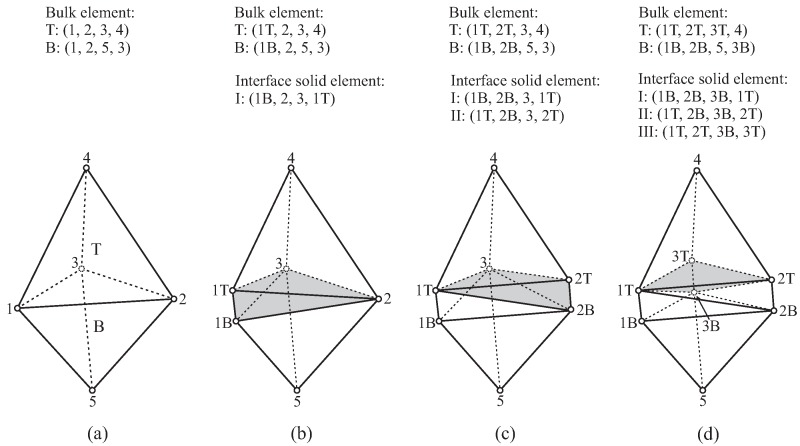
Insertion of solid elements at one interface in 3D: (**a**) original mesh with two bulk elements; (**b**) Node-1 split; ISE-I inserted (gray colored); (**c**) Node-2 split; ISE-II inserted (gray); (**d**) Node-3 split; ISE-III inserted (gray).

In Figure 11, the insertion algorithm at a 3D interface is illustrated:

1. Initially, the interface using Nodes-1, 2 and 3 is shared by two bulk elements (Element-T and Element-B).
2. When Node-1 is separated, activate the corresponding cluster of phantom nodes, including Node-1T and Node-1B. Update the nodes of bulk Element-T and Element-B. Create the first interface solid element (ISE-I).
3. When Node-2 is split, activate the phantom nodes (2T and 2B). Update the nodes of both bulk elements, as well as the existing ISE-I. Generate the second ISE (ISE-II).
4. Node-3 is activated. Similarly, the new Node-3T and Node-3B are used; the nodes of the bulk elements and the existing two ISEs (I and II) are renewed. Finally, ISE-III is created.

In analogy to the 2D situation, the scheme described above is valid for any interface in the mesh; depending on the number (equal to 0, 1 or 2) of already activated joints and the number of nodes that should be separated (1, 2 or 3), there are in total six cases possible in 3D.

Finally, it is worth emphasizing that the systematic description and maintenance of the topological and geometrical data structure of the finite element model play an important role in the implementation of an adaptive remeshing algorithm (see also [35,36,49]). The major difference between the proposed algorithm and the existing adaptation techniques for classical interface elements is that solid elements are created in the interfacial gaps, which makes it necessary to update the nodes of the existing bulk elements and, in particular, of the interface solid elements.

## 4. Numerical Examples

In this section, the performance of the proposed adaptive insertion technique for interface solid elements is demonstrated through selected numerical simulation of the crack propagation in plain and fiber-reinforced concrete structures both in 2D and 3D configurations.

### 4.1. 2D Verification Examples

The first examples are concerned with the 2D numerical simulation of plain concrete structures, conducted mainly for illustration and verification purposes.

#### 4.1.1. Illustration of Mesh Adaption Process

**Figure 12:**
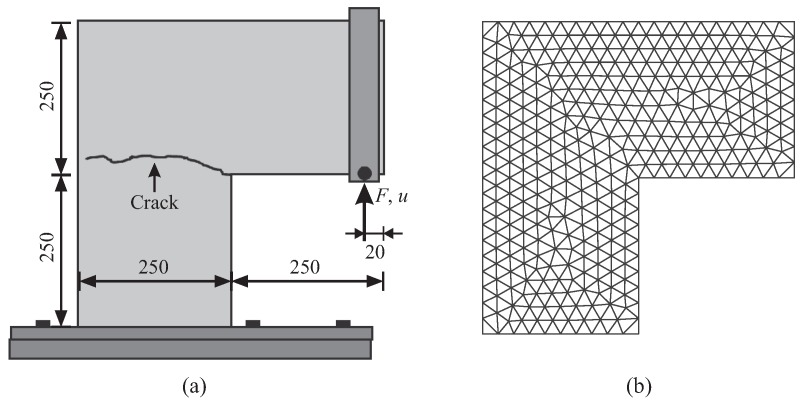
“L-shaped” panel test: (**a**) experimental setup (dimensions in mm) and crack pattern [50]; (**b**) original finite element discretization.

The “L-shaped” panel test is documented in [50] and has been selected by a few researchers to demonstrate the quality of concrete cracking models (e.g., [51,52]). The 100-mm-thick panel, made of plain concrete, is fixed at the bottom and subjected to a concentrated load, which eventually causes the failure of the panel, as shown in Figure 12a. During the test, the macroscopic crack initiates at the inner corner as a result of the concentrated tensile stresses and propagates first with a small angle with respect to the horizontal direction and proceeds almost horizontally through the panel. This test is re-analyzed via the proposed computational model using interface solid elements in a 2D configuration. The material parameters used for plain concrete are: the Young’s modulus $E_{c}=$ 25,850 MPa, the tensile strength $f_{t}=$ 2.7 MPa and the tensile fracture energy $G_{f}=$ 0.09 MPa·mm [51]. For illustration purposes, a coarse and homogeneous FE-discretization is considered for the original mesh containing 658 constant-strain-triangular elements (Figure 12b); in addition, a large value of the width of interfacial gap h=4 mm is used while separating the edges. Note that, in principle, *h* should be small, so that the model topology is not modified excessively by interfacial gaps and holes after mesh processing. On the other hand, as experience shows, a too small value of *h* may lead to numerical problems. In the present work, *h* is generally set to approximately 1/1000 of the bulk element size, based on the studies and suggestions in [18,53].

**Figure 13:**
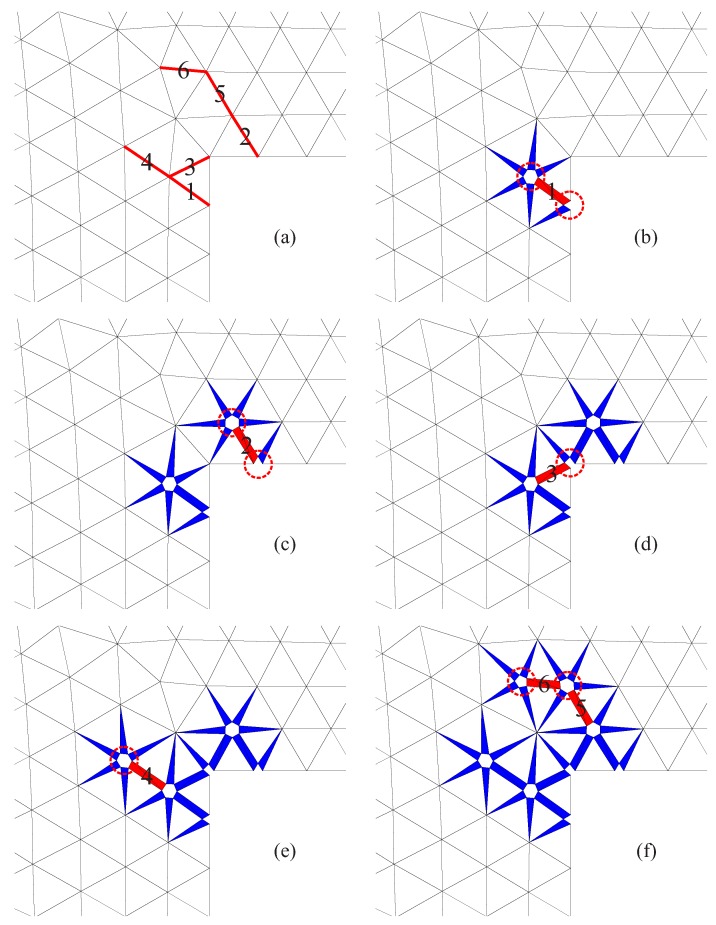
Progressive insertion of ISEs during the 2D simulation of the “L-shape” test: (**a**) original mesh (with the interfaces activated according to the labeled sequence; (**b**–**e**) splitting of Edge-1–4; (**f**) splitting of Edge-5 and -6. The red triangles represent the solid elements created at a new fully-separated interface; the blue triangles are the solid elements at partially open interfaces; the red dashed circles indicate the newly-activated joints.

As can be observed in Figure 13, as the load increases, the concentrated tensile stresses at the inner corner cause the tensile stress (traction) at the respective interfaces to exceed the threshold (Equation (12)). The interfaces are activated and split according to the sequence given in Figure 13a:

- With the activation (full splitting) of Interface-1, two nodes belonging to this edge are disintegrated; the corresponding two clusters of phantom nodes are activated, as indicated by the red dashed circles in Figure 13b. In the interfacial gap, two ISEs (triangular elements in red color) are created, while in each of the adjacent interfaces, one ISE (triangular element in blue) is inserted.
- Interface-2 is activated, where similarly, two ISEs are placed in the gap and several ISEs are generated in the vicinity (Figure 13c).
- The activation of Interface-3 leads to the splitting of only one node because the other node is already activated. At Interface-3, in addition to the existing ISE generated when Interface-1 was activated, the second ISE is created (Figure 13d).
- After the split of Edge-4 (Figure 13e), Edge-5 and Edge-6 are activated almost simultaneously.

Note that in the mesh adaptation process described above, every single interface follows the scheme introduced in Section 3.2.3.

#### 4.1.2. Results from the Adaptive Crack Model

**Figure 14:**
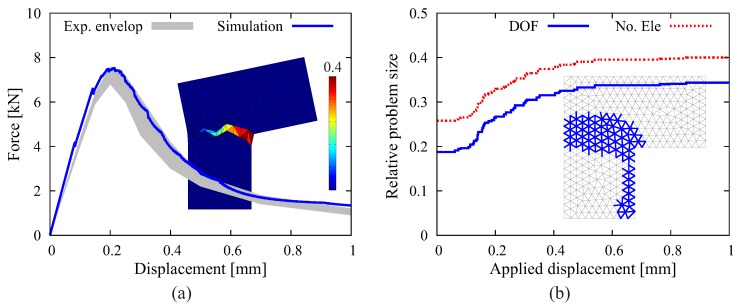
Results from the 2D simulation of the “L-shape” test using the proposed adaptive crack model: (**a**) comparison between the structural responses obtained from simulation and experiment; (**b**) evolution of the relative problem sizes (ω) regarding the system degree of freedom and number of elements. The generated interface solid elements are inserted in color in the mesh).

**Figure 15:**
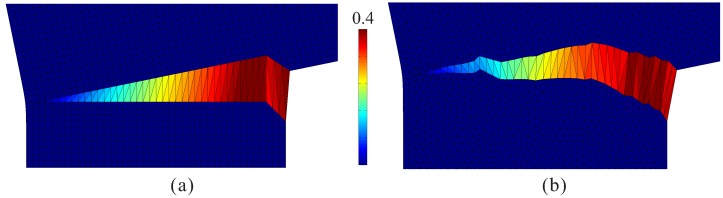
Results from the 2D simulation of the “L-shape” test using the proposed adaptive crack model: (**a**) crack pattern obtained with a structured refined mesh; (**b**) crack pattern obtained with an unstructured refined mesh.

A simulation of the L-shaped slab test is conducted by setting h=0.01 mm and γ=0.8. The results obtained from the complete simulation are contained in Figure 14. It is observed that the computed force-displacement relation, as well as the crack pattern agrees, despite the relative coarse discretization, well with the experimental data. Nevertheless, as shown in Figure 15, the quality of the crack path is improved when using particularly unstructured refined meshes [54].

In the following, the efficiency of the adaptive algorithm is discussed based on the results shown in Figure 14. The simulation starts with the original mesh, which contains approximately 19% of the number of nodes and 26% of the number of elements as compared to the case of full fragmentation; in other words, at the start of the analysis at increment i=1,

(13) $$\omega_{\mathrm{node},1}=\frac{N_{\mathrm{node},1}^{\mathrm{adp}}}{N_{\mathrm{node}}^{\mathrm{full}}}\approx 0.19,\;\omega_{\mathrm{elem},1}=\frac{N_{\mathrm{elem},1}^{\mathrm{adp}}}{N_{\mathrm{elem}}^{\mathrm{full}}}\approx 0.26.$$

Here, ω denotes the relative problem size while using the adaptive algorithm as compared with the case of full fragmentation. With the growth of the macroscopic crack, ISEs are gradually generated and located along the potential crack path. When the applied displacement reaches 0.6 mm, the propagating macroscopic crack almost penetrates the sample. Afterwards, the major crack continues to open, and the structure fails rapidly; the relative problem sizes change marginally, approaching approximately 34% for the number of nodes and 40% for the number of elements, respectively, at the end of simulation when u=1 mm at increment i=1000:

(14) $$\omega_{\mathrm{node},n_{\mathrm{final}}}=\frac{N_{\mathrm{node},n_{\mathrm{final}}}^{\mathrm{adp}}}{N_{\mathrm{node}}^{\mathrm{full}}}\approx 0.34,\;\omega_{\mathrm{elem},n_{\mathrm{final}}}=\frac{N_{\mathrm{elem},n_{\mathrm{final}}}^{\mathrm{adp}}}{N_{\mathrm{elem}}^{\mathrm{full}}}\approx 0.40.$$

Here, $n_{\mathrm{final}}$ stands for the last loading increment ($n_{\mathrm{final}}=1000$) applied during the computation. In order to provide a practical assessment of the overall efficiency of the proposed cracking model, we define the “reduction factor” Ω for a specific problem as the average relative problem size using adaptive insertion as compared to that using full fragmentation.

In this context, the reduction factors $\Omega_{\mathrm{elem}}$ and $\Omega_{\mathrm{node}}$ are computed as:

(15) $$\Omega_{\mathrm{elem}}=\frac{1}{n_{\mathrm{inc}}}\sum_{i=1}^{n_{\mathrm{inc}}}\frac{N_{\mathrm{elem},i}^{\mathrm{adp}}}{N_{\mathrm{elem}}^{\mathrm{full}}}=\frac{1}{n_{\mathrm{inc}}}\sum_{i=1}^{n_{\mathrm{inc}}}\omega_{\mathrm{node},i}\;,\;\Omega_{\mathrm{node}}=\frac{1}{n_{\mathrm{inc}}}\sum_{i=1}^{n_{\mathrm{inc}}}\frac{N_{\mathrm{node},i}^{\mathrm{adp}}}{N_{\mathrm{node}}^{\mathrm{full}}}=\frac{1}{n_{\mathrm{inc}}}\sum_{i=1}^{n_{\mathrm{inc}}}\omega_{\mathrm{elem},i}\;.$$

Referring to the diagram in Figure 14b, the reduction factor can be graphically understood as the ratio of the area under the curve to the full area (with the height = 1), which gives $\Omega_{\mathrm{node}}\approx 0.30$ and $\Omega_{\mathrm{elem}}\approx 0.36$. From the documented results, it becomes clear that a significant reduction of the total computational cost is achieved when using the proposed adaptive ISE insertion strategy as compared to inserting interface elements a priori in the entire mesh.

This simulation, coded in MATLAB${}^{®}$ (2016a, MathWorks, Natick, USA) and run on a desktop PC equipped with a quad-core i7 CPU allowing parallelization and 16 GB memory, took approximately 36 s, as compared to 107 s required for the analysis with full insertion of interface solid elements.

#### 4.1.3. Factors Influencing the Efficiency of the Adaptive Strategy

**Figure 16:**
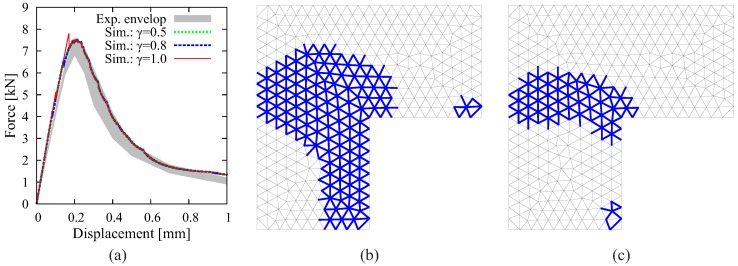
Results of the 2D simulation of “L-shape” test using the proposed adaptive crack model: (**a**) comparison between the structural responses obtained from the simulations with different values of γ; (**b**) all of the created interface solid elements (blue lines) for the case of γ=0.5; (**c**) all of the created interface solid elements (blue lines) for the case of γ=1.0.

Evidently, the efficiency of the adaptive insertion technique highly depends on the specific problem and the computational settings. Figure 16 illustrates the influence of the stress threshold factor (γ) for the determination of critical interfaces (see Equation (12)). Adopting a small value of γ=0.5 yields the same simulation results as γ=0.8 used in the previous calculation. However, a significantly larger number of interface solid elements is created. With a high value (γ=1.0), although less ISEs are generated, the over-stressing phenomenon due to the delayed processing of interfaces is observed in Figure 16a. It is noted that the choice of γ depends on the load incrementation; larger increments require a smaller value of γ.

**Figure 17:**
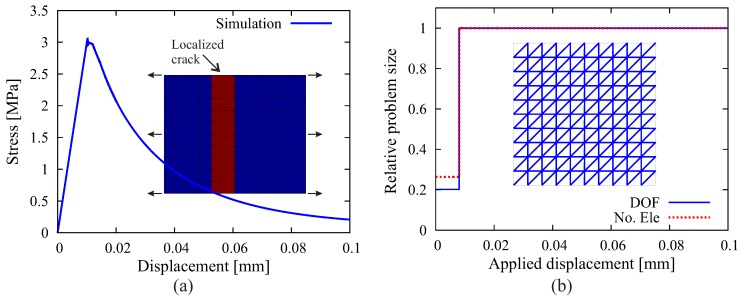
Results of the 2D simulation of uniaxial tension on a square specimen: (**a**) computed structural responses and crack pattern; (**b**) evolution of the relative problem sizes regarding the system degree of freedom and number of elements, as well as all of the created interface solid elements (blue lines in the mesh).

Another aspect discussed next is the influence of stress distribution. A test is performed, characterized by a square panel subjected to uniaxial tension (Figure 17a). This simple case is often considered for the verification of cracking models for quasi-brittle materials and for the assessment of the influence of different model features, such as the representation of the microstructure and the heterogeneous fracture characteristics of concrete [55,56]. The same material parameters as for the “L-shaped” panel are assumed. As expected, a localized crack splitting the panel develops and leads to the failure of the specimen (Figure 17a). In Figure 17b, one can clearly see that in such an extreme situation characterized by a uniform distribution of tensile stresses in the domain, all interfaces reach the critical state simultaneously at the level of applied displacement 0.08 mm; consequently, the complete mesh is fragmented and filled with interface solid elements. Hence, in the case of a homogeneous stress distribution, the advantage of the adaptive algorithm as compared to a full insertion of ISEs vanishes. In practical situations, however, crack propagation is accompanied with high stress concentrations, where the adaptive algorithm performs excellently as shown in the previous example of the “L-shape” test.

### 4.2. Application to 3D Structural Analyses

The following examples are concerned with the performance of the implemented adaptive insertion technique for interface solid elements in 3D analyses. In all of these examples, γ=0.8 and h=0.01 mm are used for generating the ISEs.

#### 4.2.1. Plain Concrete Notched Beam under Bending

**Figure 18:**
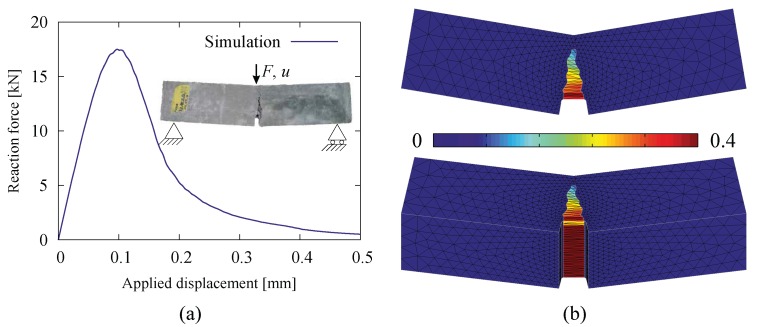
Results from a 3D simulation of the notched FRC beam made of plain concrete: (**a**) load-displacement diagram; (**b**) crack pattern (side view and bottom-side view with the contours representing the crack opening magnitude in the deformed configuration).

A notched beam subjected to three-point bending (Figure 6) is re-analyzed. The beam has a length of 600 mm, a height of 150 mm and a width of 150 mm. A notch with a depth of 30 mm is located at the bottom side at the mid-span of the beam. A total displacement of 0.5 mm is applied incrementally at the mid-span on the top surface, which leads to the failure of the beam induced by a vertical crack initiating at the notch tip and propagating towards the top surface (Figure 18).

The original 3D FE-discretization contains 6743 nodes and 34,537 linear tetrahedral elements. For comparison, the simulation is first performed with full insertion of ISEs, which requires the completely fragmented mesh filled with three solid elements at every interfacial gap; consequently, the preprocessed mesh includes 138,148 nodes (approximately 20-times that of the original mesh) and 235,939 elements (approximately seven-times that compared to the original mesh), among which, 201,402 are ISEs.

In a first analysis, the beam is assumed to be made of plain concrete, and in a second analysis, a fiber-reinforced concrete beam is re-analyzed and compared with experimental results. This has been analyzed by the authors as a 2D problem without using an adaptive strategy for the ISE insertion in [17]. The simulation results for the plain concrete beam, including the load-displacement curve and the crack pattern, are shown in Figure 18.

**Figure 19:**
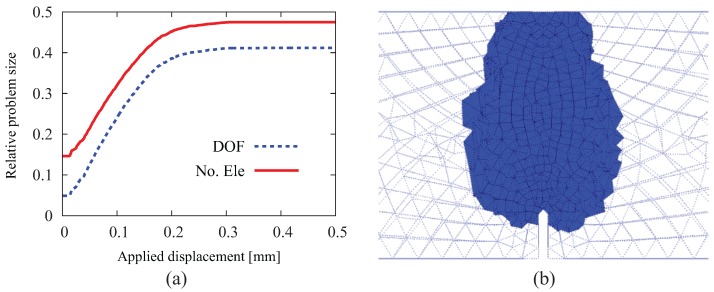
Results from a 3D simulation of the notched FRC beam with adaptive insertion of ISEs: (**a**) evolution of the relative problem sizes in terms of the number of degrees of freedom and the number of elements, respectively; (**b**) generated ISEs at the end of the simulation (u= 0.5 mm).

By employing the adaptive insertion technique, simulation results are obtained without any noticeable difference. However, the computation starts with the original mesh with interface solid elements being first inserted in the vicinity of the notch-tip and then continuously added in the vicinity of the front of the macroscopic crack, which propagates vertically into the beam until the structure fails. At the end of simulation (u=0.5 mm), only 77,538 ISEs are created and distributed along the structural crack (Figure 19b). As compared to the case of full insertion, the efficiency of adaptive insertion is clearly seen in Figure 19a. At the beginning, only 5% of the system degrees of freedom (DOF) and 15% of the number of elements are used for the computation; these values increase with the loading procedure. When the structural crack approaches the top surface (u> 0.3 mm), the insertion of new ISEs in every load step becomes negligible, and the problem size remains more or less constant at the level of approximately 41% in regard to the system DOFs and 48% in regard to the number of elements. As a general assessment, the reduction factors are evaluated according to Equation (15) as $\Omega_{\mathrm{elem}}=0.44$ and $\Omega_{\mathrm{DOF}}=0.37$ (identical to $\Omega_{\mathrm{node}}$).

#### 4.2.2. Fiber-Reinforced Concrete Notched Beam under Bending

**Figure 20:**
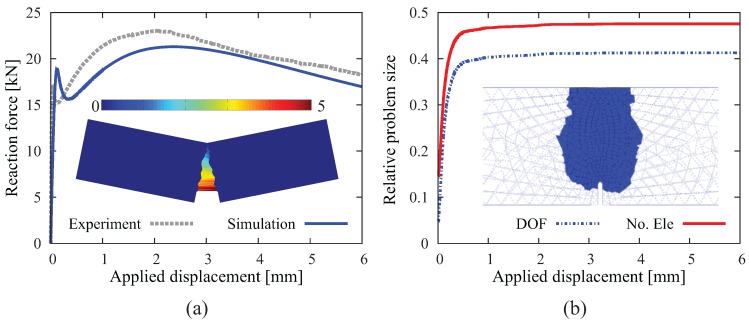
Results of a 3D simulation of the fiber-reinforced notched beam test: (**a**) comparison of load-displacement curves obtained from the adaptive finite element analysis and from the experiment [41] and crack pattern; (**b**) evolution of the relative problem sizes in terms of the number of degrees of freedom and elements, respectively, and generated ISEs at the end of simulation (u= 6 mm).

In the second analysis, the same beam geometry is adopted, however, now assumed to be made of fiber-reinforced concrete with 60 kg/m${}^{3}$ long hooked-end fibers. Figure 20a shows the load-displacement relation obtained from the numerical simulation (full line). The comparison with the experimental result (dotted line) shows a good agreement over the complete loading range. The figure also contains the crack pattern as the contour plot of the crack opening magnitude in the deformed configuration (with 10-fold magnification of displacement). The efficiency of the adaptive insertion technique is clearly shown in Figure 20b. In the early stages of load (u< 0.3 mm), due to the quick advance of the macroscopic crack, the relative problem size in regards to the number of degrees of freedom and the number of elements grows rapidly. After the major crack surface approaches the top surface of the beam, the structure exhibits a rather ductile behavior with considerable residual load-bearing capacity as the crack continues to open and the hooked-end steel fibers intercepting the crack are successively activated and eventually pulled out from the concrete matrix. In this loading stage, the relative problem size remains at a certain level without significant change. The simulation proceeds until u= 6 mm due to the fairly ductile behavior of the FRC specimen. The reduction factors are evaluated in this case as $\Omega_{\mathrm{elem}}=0.46$ and $\Omega_{\mathrm{DOF}}=0.40$.

#### 4.2.3. Plain and Fiber-Reinforced Concrete Notched Beam Subjected to Torsion

The last example qualitatively demonstrates the model performance in the full-3D situation that cannot be simplified to 2D. To this end, the so-called “Brokenshire test”, where, by imposing torsion onto a concrete prism with an inclined notch, a non-planar crack surface develops [57], is re-analyzed numerically. The original test on the plain concrete specimen has been replicated by the previous version of the model (see [17] for more details); similar validations have been performed applying different cracking models including the smeared, embedded and discrete approaches [37,58,59]. Here, the computational simulation of the plain concrete beam test is re-conducted using the adaptive crack model. In a second analysis, we assume, that the beam is made of fiber-reinforced concrete, with 60 kg/m${}^{3}$ short straight fibers being added into the concrete matrix.

**Figure 21:**
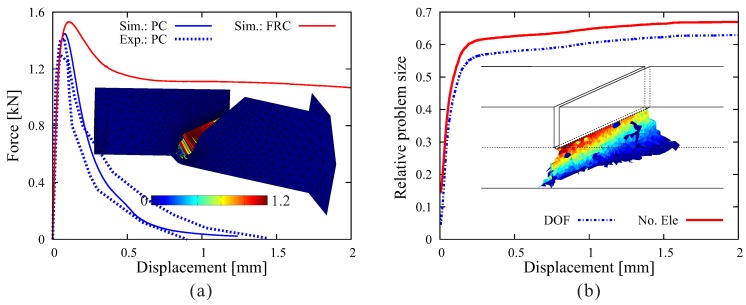
Results of a 3D simulation of a plain- and a fiber-reinforced notched prism subjected to torsion: (**a**) load-displacement curves obtained for plain- and fiber-reinforced concrete, respectively, and the crack pattern for the FRC prism; (**b**) evolution of the relative problem sizes in terms of the number of degrees of freedom and elements, respectively. The inlet contains the twisted crack surface obtained from the analysis of the FRC prism.

The original mesh contains 8119 nodes and 42,427 linear tetrahedral elements. Figure 21a shows the load-displacement relation obtained for the FRC prism (full red line). Comparing this result with the result from the plain concrete specimen (full blue line), a considerable increase of the ductility is observed. Experimental data are only available for the plain concrete case and not for the FRC prism. Figure 21a contains the range of the experimental load-displacement curves for plain concrete documented in [57] as dotted lines. An excellent agreement between the computed and the measured load-displacement curves is observed. It is noted that according to the numerical simulations, the crack pattern does not differ significantly between the plain concrete and the FRC prisms. In both cases, a non-planar crack develops (see also the inlet in Figure 21b). In regard to the efficiency of the adaptive insertion technique (Figure 21b), a similar tendency as found in the previous example of the 3D analysis of FRC beam is noticed (see Figure 20b): in the early stage, the relative problem size grows rapidly and becomes almost stationary after the first progressive growth of the crack. The simulation ends with a mesh possessing 106,875 nodes and 194,923 elements. The reduction factors for this simulation are evaluated as $\Omega_{\mathrm{elem}}=0.56$ and $\Omega_{\mathrm{DOF}}=0.50$. It is noticed that, as compared to the previous example, the efficiency of the adaptive strategy is less pronounced in this example; this is due to the a priori refined mesh in the local region of the notch. Nevertheless, in regard to the computation time, this simulation took 6.5 h, which is only 23% of the time (28.1 h) required for the analysis using full ISE insertion!

## 5. Concluding Remarks

A multilevel modeling framework previously developed for the analysis of fiber-reinforced concrete was implemented in an adaptive computational crack model using continuum interface solid elements (ISEs) for 2D and 3D simulations of fiber-reinforced concrete structures. The ISE formulation allows one to capture complex fracture processes without the need for a crack tracking algorithm. Since the numerical simulation of crack propagation using interface elements usually involves a considerably larger number of degrees of freedom as compared to other classes of concrete cracking models, such as smeared crack models or embedded crack models, an adaptive procedure was proposed to perform structural crack propagation analyses with an acceptable computational effort. In contrast to the naive implementation, where the entire FE mesh is fragmented a priori and all available interfaces are filled with degenerated solid elements prior to the computation, the interface solid elements are now inserted only along those interfaces during the incremental analysis, which are identified as locations of the potential crack path according to a stress criterion. Hence, ISEs are adaptively created in the vicinity of the advancing crack front. The structural simulation starts with the original discretization of the domain using standard solid finite elements. During the failure analysis, depending on the fracture advancement, the problem size increases gradually as ISEs are inserted. The numerical examples have shown that in spite of this increase in computational costs due to mesh adaptation as the loading increases, the adaptive algorithm allows one to significantly reduce the total computational cost without affecting the simulation accuracy. In selected 2D, as well as in 3D applications, the maximum number of degrees of freedom, required only when the fracture process has advanced almost to the state of structural failure, was reduced to approximately 30–40% as compared to an analysis, in which ISEs are a priori inserted along all element edges of the discretized structure. As a consequence, the computation consumes only 15–30% of the time (according to the selected cases for comparison). In large-scale analyses, these savings in time, in association with the considerable reduction in memory requirements, may be the pre-requisite to be able to perform this type of failure analysis, if no high performance hardware environment is available. It was also noticed that the performance of the proposed adaptation technique highly depends on the characteristics of specific problem. The efficiency of the algorithm is higher in problems characterized by high stress concentrations, which is usually the case during the evolution of bending cracks.
